# Integrating Urban Adolescent Mental Health Into Urban Sustainability Collective Action: An Application of Shiffman & Smith’s Framework for Global Health Prioritization

**DOI:** 10.3389/fpsyt.2020.00044

**Published:** 2020-02-20

**Authors:** Lauren E. Murphy, Helen E. Jack, Tessa L. Concepcion, Pamela Y. Collins

**Affiliations:** ^1^ Department of Psychiatry and Behavioral Sciences, University of Washington, Seattle, WA, United States; ^2^ Department of Internal Medicine, University of Washington, Seattle, WA, United States; ^3^ Department of Global Health, University of Washington, Seattle, WA, United States

**Keywords:** adolescents, urban health, mental health, sustainable cities, global development, social determinants, sustainable development goals, health policy

## Abstract

The majority (55%) of the world’s population lives in urban environments. Of relevance to global mental health, the rapid growth in urban populations around the world and the attendant risks coincide with the presence of the largest population of adolescents the global community has seen to date. Recent reviews on the effects of the urban environment on mental health report a greater risk of depression, anxiety, and some psychotic disorders among urban dwellers. Increased risk for mental disorders is associated with concentrated poverty, low social capital, social segregation, and other social and environmental adversities that occur more frequently in cities. To address these problems, urban adolescent mental health requires attention from decision makers as well as advocates who seek to establish sustainable cities. We examine opportunities to increase the prominence of urban adolescent mental health on the global health and development agenda using Shiffman and Smith’s framework for policy priorities, and we explore approaches to increasing its relevance for urban health and development policy communities. We conclude with suggestions for expanding the community of actors who guide the field and bridging the fields of mental health and urban development to meet urban adolescent mental health needs.

## Introduction

The United Nations (UN) New Urban Agenda (NUA) was adopted in Quito, Ecuador in 2016 at the UN Conference on Housing and Sustainable Development (Habitat III) (1). The Agenda’s writers envisioned a future for cities in which all city dwellers “without discrimination of any kind are able to inhabit and produce just, safe, healthy, accessible, affordable, resilient and sustainable cities and human settlements to foster prosperity and quality of life for all (1).” The document embraces the 2030 Agenda for Sustainable Development and other relevant frameworks for development, climate change, and poverty reduction (2), and it is timely. As of 2018, 55% of the global population lived in cities, and 1 in 8 urban residents lived in a city with more than 10 million people (3). Cities offer greater opportunities for wealth, employment, social freedoms, and education but inequality is increasingly one of the hallmarks of large cities in high-, middle-, and low-income countries (4). Rapidly urbanizing cities in lower income countries struggle with insufficient urban services, from water and sanitation to healthcare and housing, but the sufficiency of these services can vary considerably within cities at any income level (4). When infrastructural resources are scarce, cities accumulate additional vulnerabilities, e.g., to climate change, to natural disasters, and the effects are most devastating for the poorest residents. In these conditions, poor mental health is one of many negative outcomes, but one of the most disabling for young people.

An estimated 13%–15% of adolescents (10–19 year olds) in low- and middle-income countries (LMIC) live with a mental disorder (5), and depression and anxiety are among the 10 leading causes of disability for children and adolescents globally (6). In the current context of global urbanization, history’s largest population of adolescents is transitioning into adulthood (7, 8). Around 1.2 billion adolescents between the ages of 10 and 19 constitute 16% of the world’s population (8). The majority of these young people live in Asia, and though less numerous in total numbers, adolescents make up 23% of the Africa region’s population (8). These two continents account for 90% of expected urban growth of the coming decades (3). Adolescents who live in cities are exposed to the risks and benefits of urban life during this dynamic and sensitive period of social and neurodevelopment and to the consequent mental health outcomes.

Generally, urbanicity increases risk for psychosis (9–11), anxiety disorders (12), and depression (12). Social isolation, neighborhood poverty, and discrimination also contribute to poor mental health (13). Despite the morbidity associated with mental disorders, neither treatment nor mental health promotion for adolescents has achieved global public health priority. Financing to address these services is distressingly low. From 2007 to 2015, around $190 million of Development Assistance in Child and Adolescent mental health was invested, which amounts to about 0.01% of development assistance for health (14). Fifteen years ago, Sclar et al. (2005) pointed to the growing population of urban adolescents and the dearth of research regarding their health needs (15). As the global community seeks to achieve targets for sustainable development that could address these needs, the time is right for articulating how diverse constituencies can meet shared goals, such as for adolescent mental health and urban sustainability (16, 17).

Urban sustainability, as defined by urban planners and envisioned by the NUA, should enable cities to better meet the mental health needs of young people. It’s goal is to “promote and facilitate the long-term well-being of people and the planet through efficient use and management of resources while improving a city’s livability, through social amenities, economic opportunity and health, and enabling the city to integrate well with local, regional and global ecosystems (18).” The guiding principles of the New Urban Agenda support this goal (**Box 1**). The World Health Organization (WHO) argues that healthy citizens are the most important asset of any city, and that “a health lens” should inform the NUA (19). We propose that full implementation of the NUA could, if properly directed, create environments that yield improved mental health for urban adolescents globally, primarily by reducing upstream or “distal” risk factors for mental disorders (16). Consequently, as decision-makers achieve their agreed upon priorities (e.g., meeting targets for the SDGs and NUA) they could also facilitate urban adolescent mental health and wellbeing.

Box 1 **Principles of the New Urban Agenda (1).**

1. Leave no one behind, by ending poverty in all its forms and dimensions, including the eradication of extreme poverty, by ensuring equal rights and opportunities, socioeconomic and cultural diversity, and integration in the urban space, by enhancing liveability, education, food security and nutrition, health and well-being, including by ending the epidemics of AIDS, tuberculosis and malaria, by promoting safety and eliminating discrimination and all forms of violence, by ensuring public participation—providing safe and equal access for all, and by providing equal access for all to physical and social infrastructure and basic services, as well as adequate and affordable housing;
2. Ensure sustainable and inclusive urban economies by leveraging the agglomeration benefits of well-planned urbanization, including high productivity, competitiveness and innovation, by promoting full and productive employment and decent work for all, by ensuring the creation of decent jobs and equal access for all to economic and productive resources and opportunities and by preventing land speculation, promoting secure land tenure and managing urban shrinking, where appropriate;
3. Ensure environmental sustainability by promoting clean energy and sustainable use of land and resources in urban development, by protecting ecosystems and biodiversity, including adopting healthy lifestyles in harmony with nature, by promoting sustainable consumption and production patterns, by building urban resilience, by reducing disaster risks and by mitigating and adapting to climate change.

The implementation of a comprehensive agenda requires prioritization and negotiation around which aspects will be fully implemented and resourced. Raising the profile of activities relevant to adolescent mental health may require specific strategies. In this *Perspective*, we apply Shiffman and Smith’s criteria for the determinants of political priority to urban adolescent mental health in the global context, in order to identify opportunities for expanding cross-sector engagement to support and prioritize adolescent mental health (20). Briefly, Shiffman and Smith assert that global health initiatives are more likely to receive political priority when four foundational factors support each other: (1) coherent internal framing of *ideas* as well as external framing of the ideas that resonate with leaders; (2) *issue characteristics* that are amenable to credible indicators, objective measurement of severity, and effective interventions; (3) favorable *political contexts* that allow actors to influence decision makers or governance structures that enable collective action; and (4) strong and unified *actor power* (20).

### Exploring Factors for Collective Action

#### Ideas: Summarizing the Internal Frame

Shiffman and Smith (2007) identify the framing of ideas about an issue as a key component of successful collective action, noting that “any issue can be framed in several ways….Some frames resonate more than others, and different frames appeal to different audiences (20).” Coherent internal framing among key actors in a given field enables the clear communication of priorities of the field to external stakeholders and facilitates unified mobilization of the field. A long history of research on adolescent development and mental health brought together in recent impactful publications demonstrates a coherent internal framing with validity for high-, middle-, and low-income settings (7, 21). Key elements of this consistent internal framing are that (1) adolescence is a dynamic period of neurodevelopment during which young people acquire higher-level cognitive, emotional, and social skills for functioning and expanded interactions beyond the home (7, 22, 23); (2) adolescence provides a meaningful opportunity to remediate insults from early life while setting healthy trajectories for adult life, and, consequently, for the next generation (21); and (3) central to adolescents’ health and wellbeing are their interactions with the physical environment, the social context, and the people in these environments (23).

#### Ideas: Options for External Framing

The “external frame,” is the way an issue is portrayed to policymakers and the public (20). We present four ways of framing adolescent mental health that intersect with the principles of the New Urban Agenda: a social justice frame, an economic frame, an urban design and intersectoral governance frame, and a security frame.

Adolescents are dying from preventable causes associated with their mental health, and they have a right to mental health care. Suicide is the second leading killer of older adolescents (24), and the prevalence of self-harm and suicidal behaviors is as high as 15%–31% and 3%–4.7%, respectively, among adolescents in LMICs (25). Self-harm elevates risk of future suicide 30–100 fold (26, 27). The Universal Declaration of Human Rights and the Declaration of the Rights of the Child, which include the rights to health, education, and freedom from neglect, codify adolescents’ rights to treatment of mental illness and promotion of mental health (28, 29). Using a human rights lens broadly appeals to the global community (30), which may be important for adolescent mental health advocates, and could serve a unifying purpose as they seek to influence health, education, and urban planning sectors. As Shiffman and Smith show in their case study of the successful elevation of maternal health on the global agenda, a social justice frame added urgency to the issue and spoke to the public, in addition to content experts, which helped build support (30).

“Investments in adolescent health and wellbeing [including mental health] bring a triple dividend of benefits now, into future adult life, and for the next generation of children” (7), and investments in urban neighborhoods are an investment in adolescents. Indirect investments through poverty reduction strategies that increase access to quality education, food security, and mental health services should improve adolescent mental health while also supporting the principles of urban sustainability. Concentrated poverty in urban neighborhoods contributes to poor mental health outcomes (e.g., hopelessness, anxiety, and depression) (31, 32). Direct investments in urban mental health services compound benefits as treatment of mental illness has been shown to improve economic outcomes (33). Highlighting the economic costs of poor mental health or benefits of promoting mental health and averting or treating mental illness may speak to policymakers’ needs to seek significant returns on investment and reduce spending over the long-term (34).

Data from South Africa (35), Kenya (36), as well as India, Nigeria, South Africa, and China (37) show that even as urban communities await the implementation of successful poverty reduction strategies or investments in health and education, other factors can support adolescent mental health. Intersectoral urban polices and thoughtful urban design create additional paths to adolescent mental health. Social protection policies that reduce maltreatment and improve parental caregiving quality, and community structures that permit quality peer relationships can support resilience for children with multiple stressors (35). Cultivating friendly home environments, ensuring parental presence in the home, and supporting parental monitoring can reduce behavioral risk even in the most distressed settings, such as informal settlements (36). Community design that enhances neighborhood social support and connection could promote adolescent mental health in some African and Asian contexts (37). Locally accessible economic opportunities that provide resources to adult female carers could be of particular benefit to adolescent girls (37).

A security frame may have less broad appeal, but particular salience for leaders in urban environments racked by youth violence or terrorism. Violence can be both a cause and consequence of poor mental health. Social cohesion, low social control, high neighborhood disorder, and violent victimization or fears of it are associated with adverse adolescent mental health outcomes in high-income countries (11, 31, 32, 38). Growing up in areas with high violence and low social support has been associated with use of negative psychological coping mechanisms, such as acting out or aggressive behavior (39, 40). In communities affected by violence and poverty, considerable international resources are being devoted to the prevention of radicalization (41, 42) and many of these efforts target adolescents (43). A recent report by the UN Development Program used qualitative interviews with people in Africa who were recruited to violent extremist groups to better understand what drew them to those groups (43). Respondents noted that unhappiness in childhood and poor parental support contributed to their participation in extremist groups, and the UNDP called for programming to support healthy parenting, outreach to high risk youth, and initiatives to promote staying in school (43). Anti-radicalization programs may bring unprecedented resources, but come with the ethical complexity of working within defense budgets. Youth mental health advocates should avoid the stigmatizing and dehumanizing framing of adolescents as potential threats to security, but may be valuable voices in efforts to prevent violence and radicalization by strengthening family and educational systems.

#### Issue Characteristics

The perceived severity of an issue, the availability of credible indicators, and the perceived effectiveness of solutions also determine its political priority (20). Challenges for the perceived severity of child and adolescent mental health problems in LMICs include a small, albeit growing, evidence base, limited awareness of youth mental health issues, and scarce human resources to address these issues (44). In the absence of specialists, clarity of identification and diagnosis create additional challenges to ascertaining illness. However, data do exist to demonstrate the high prevalence of self-injurious and suicidal behaviors among adolescents in LMICs—markers of severity. The Global Burden of Disease studies play a valuable role in providing credible indicators for mental disorders by estimating the prevalence and the disease burden of adolescent mental disorders in HICs and LMICs (6, 45–47). UNICEF’s initiative, Measurement of Mental Health among Adolescents at the Population Level (MMAP) seeks to validate instruments in diverse cultural contexts (48). The Lancet Commission on adolescent health and wellbeing now tracks 12 indicators for progress in adolescent health and wellbeing (7, 49). These capture disability adjusted life years for communicable, maternal, and nutritional disorders; injury and violence; and non-communicable diseases, including mental and substance use disorders (7, 49). They also track health risks, such as smoking, binge drinking and obesity as well as social determinants of health (i.e., educational attainment, birth rates, marriage age, the proportion of older adolescents and youth who are not in employment, education or training (NEET) (49). Such indicators can also serve as milestones for achieving urban sustainability. Importantly, feasible, effective interventions for implementation in urban settings can address risks for poor mental health (**Table 1**).

**Table 1:** The New Urban Agenda and elements that support adolescent mental health: mental health sequelae and interventions.

NUA vision for cities	Elements that support adolescent mental health and wellbeing	Mental health sequelae	Relevant Interventions
Cities fulfill their social function, including right to housing and an adequate standard of living, access to drinking water and sanitation, food security and nutrition, health, education, mobility, energy, air quality, livelihoods	Access to adequate housing	Adequate housing associated with improved mental health,(50–54) while lack of housing associated with poor mental health (55–57).	Mental Health Promotion among adolescents in schools using life skills education (58) Supporting Adolescent Orphan Girls to Stay in School (59) Community-based skills-development programs for girls(60)
	Food security and nutrition	Lack of food security and poor nutrition are associated with psychological distress, (61) increased exposure to violence, (62) suicidal behavior, (63) and poor mental health (64–66) including anxiety and depression (67).	
	Education	Quality education supports cognitive development and mental health (52, 68, 69)	
	Health	Timely access to health services enable early identification and management of mental health problems (70–73)	
Cities are participatory, promote civic engagement, engender a sense of belonging and ownership, foster social cohesion, prioritize safe, inclusive quality public spaces	Promote participatory engagement	Adolescence is a time for building agency, and youth civic engagement is positively associated with mental health into adulthood (74–77).	School-based life skills training (78) Social Networking Action for Resilience (79) Family-centered programing (80) Social identity intervention to build and strengthen social group membership (81) Social connectedness in street-involved youth (82)
	Social cohesion and belonging	Social connectedness (peers, school, family) promotes adolescent mental health (80–83).	
	Safe public spaces	Safe public spaces are essential for adolescent development, socialization, and mental health (84).	
Cities achieve gender equality and empower all women and girls and prevent discrimination, violence, and harassment	Reduced exposure to gender-based violence	Adolescents, particularly young women, who experience gender-based violence are at higher risk of poor mental health outcomes (85, 86)	Intimate partner violence prevention (87, 88) Gender-based violence prevention program (89, 90)
	Reduced exposure to gender-based discrimination	Gender-based discrimination has negative effects on youth mental health (91).	
Cities meet the challenges and opportunities of inclusive and sustained economic growth	Reduce youth unemployment	Youth employment has positive effects on both youth mental health (90, 92–94) and economic growth	Economic empowerment intervention (88) Vocational training (95, 96)
Promote age- and gender-responsive planning and investment for sustainable, safe accessible urban mobility	Increased urban mobility for youth	A built environment which enables safe transport and mobility has positive impacts on youth mental health (97–99).	Transport-related factors that could impact public mental health (100)

### Actor Power

The ideas, evidence and framing of urban adolescent mental health needs can only be meaningful when conveyed and sustained by an influential group of actors and institutions—the policy community (101). For global mental health and adolescent health, this community continues to grow in size and diversity. These actors have helped to create and support a set of rules, norms and strategies within and across organizations that help promote portrayals (or framing) of adolescent health and wellbeing (101, 102). The launch of WHO’s Global Accelerated Action for the Health of Adolescents (AA-HA)!, the Global Strategy for Women’s Children’s and Adolescents’ Health, and the Lancet Commission on adolescent health and wellbeing (7), and UNICEF’s and other multilateral interests in the intersections of gender, mental health, and adolescence (103), reflect the increasing prominence of adolescent health on the global health agenda and the action of the policy community. Guiding institutions in global mental health (e.g., the World Health Organization, the Lancet Commission on Global Mental Health and Sustainable Development) also highlight adolescent mental health as priority (17).

Importantly, additional actors have joined the policy community. The voices of people with lived experience of mental health conditions are present. Youth advocates have also joined the community (https://globalmentalhealthcommission.org/youth-campaign/). There remain critical missing voices. A majority of the published research we identified on urban adolescent mental health focused on young people in distressed communities in HICs and LMICs. These youth represent a resource for engagement in advocacy for urban mental health. The New Urban Agenda identifies youth engagement and capacity development as explicit goals (1), and there is preliminary evidence that involvement in collective action can promote youth development among adolescents in some contexts (74). Global platforms like citiesRISE, which value intersectoral collaboration along with youth mobilization and leadership for progress in adolescent mental health, could help to bridge these policy communities (104).

In order to bridge relevant communities, the global mental health community and child and adolescent advocates need to learn the language of urban sustainability and generate knowledge relevant to both sectors. Universities, which often support and foster interdisciplinary collaborations, may be ideal settings for engaging young people and bridging urban design, mental health, climate, public health, and other relevant constituencies. New publications and recent textbooks (105) are also establishing this field as a discipline (https://www.urbandesignmentalhealth.com/), which will help to strengthen the evidence generated from new collaborative efforts.

### Policy Contexts

Shiffman and Smith argue that one route to political priority in global health is to take advantage of policy windows, i.e., political moments that present opportunities for advocates to influence decision makers (20). The NUA and the SDG’s provide such opportunities for advocates of urban adolescent mental health. Many non-health SDGs are associated with distal determinants of poor mental health and achieving certain SDG targets could provide particular benefit for urban adolescents, given the relationships among environment, social experience and mental health outcomes (16). Together, these documents provide a platform for integrating adolescent mental health within urban development.

While international agreements are vital, domestic policy windows provide opportunities for small steps toward integration of adolescent mental health into the broader health and development agenda. Zimbabwe’s Ministry of Health and Child Care recently formed a Mental Health Research Task Force to set national research priorities and better coordinate and publicize research (106). While the Task Force includes a diverse set of researchers and practitioners, including some with an interest in child and adolescent mental health, it includes no one who focuses on urban planning or city development. Especially at the outset of the taskforce, there are opportunities to advocate for the inclusion of new perspectives. Finding opportunities to incorporate voices of mental health specialists into urban planning conversations and urban planners into mental health conversations may help advance adolescent urban mental health in local or national policies and priorities.

In addition to policy windows, Shiffman and Smith draw attention to global governance structures (norms and institutions), which can both facilitate and impede collective action. Responsibility for adolescent mental health in an urban environment spans numerous areas of government, including ministries of health, urban planning, and criminal justice institutions. This fragmentation of responsibility can lead to “buck passing”, in which every institution believes that the problem is the responsibility of another institution, and thus no institution takes definitive action (30, 107). Advocates face the challenge of uniting these institutions around common commitments and projects to promote adolescent wellbeing. Conversely, the benefit of having multiple institutions involved is that there is potential for pooling of resources toward a common goal (108). While it does not focus on adolescents, one model of successful cross-sectoral collaboration between health and urban planning is the Research Initiative for Cities and Health (RICHE) network, an interdisciplinary group based out of University of Cape Town that has set priorities for and works to promote urban health research in Africa (109, 110).

How and whether decision makers seize policy windows to promote action for mental health is also influenced by the cultural context. In settings where tremendous social stigma is attached to mental disorders and where structural stigma relegates mental health issues to low priority, identifying and vocally advocating for those portions of other agendas (like the NUA or SDG 11) that can enhance mental health outcomes becomes even more important.

### Other Considerations

We have outlined how Shiffman and Smith’s criteria–the framing of ideas, engaging an enlarged policy community, using evidence to shape the messages, and taking advantage of the current political context, i.e., countries’ desires to achieve the SDGs—can contribute to prioritization of actions that simultaneously support adolescent mental health and action for urban sustainability (20). Shiffman notes that these factors lack a theoretical basis, but the importance of ideas and how they are portrayed, as well as the strength of the institutions the policy community forms, become more salient factors of influence through some theoretical lenses (101). Perspectives of mental health advocates in an LMIC setting support the importance of coordinating a shared message among many stakeholders, active engagement with decision makers, and effectively communicating messages (111).

## Conclusions

Chisholm and colleagues showed that investment in mental health in LMICs yields a significant return (112). Investment in adolescent mental health is particularly advantageous. Adolescence is a critical stage of development, and the contexts in which adolescents live help shape their ability to contribute fully to society socially, economically, and intellectually. Importantly, the actions to achieve these ends can be integrated with other efforts. Just as integrating mental health into broader health care efforts is critical for achieving good outcomes for most health conditions, (113, 114), what we know about urban adolescent mental health can inform implementation of the New Urban Agenda. In order for this to occur, relevant decision-makers must recognize the value and importance of actions that support adolescents in urban contexts. Progress toward translating strategies to action that successfully influences decision makers will depend on meaningful alliances across sectors and disciplines, careful understanding of political leaders’ concerns in local contexts (101), and the ability to thoughtfully and nimbly respond to these.

## Author Contributions

PC developed the concept of the manuscript. LM, TC, HJ, and PC reviewed relevant literature, and wrote sections of the manuscript. PC and HJ wrote the final draft. All authors approved the final manuscript.

## Funding

PC was supported in part by a contract with the Global Development Incubator for research coordination activities with citiesRISE.

## Conflict of Interest

The authors declare that the research was conducted in the absence of any commercial or financial relationships that could be construed as a potential conflict of interest.
